# Antibiotic-Resistant Septicemia in Pediatric Oncology Patients Associated with Post-Therapeutic Neutropenic Fever

**DOI:** 10.3390/antibiotics8030106

**Published:** 2019-07-30

**Authors:** Rosalino Vázquez-López, Omar Rivero Rojas, Andrea Ibarra Moreno, José Erik Urrutia Favila, Adan Peña Barreto, Guadalupe Lizeth Ortega Ortuño, Jorge Andrés Abello Vaamonde, Ivanka Alejandra Aguilar Velazco, José Marcos Félix Castro, Sandra Georgina Solano-Gálvez, Tomás Barrientos Fortes, Juan Antonio González-Barrios

**Affiliations:** 1 Departamento de Microbiología del Centro de Investigación en Ciencias de la Salud (CICSA), FCS, Universidad Anáhuac México Campus Norte, Cuidad de México 52786, Mexico; 2 Coordinación Ciclos Clínicos, Facultad de Ciencias de la Salud, Universidad Anáhuac México Campus Norte, Cuidad de México 52786, Mexico; 3 Departamento de Microbiología y Parasitología, Facultad de Medicina, Universidad Nacional Autónoma de México, Ciudad de México 04510, Mexico; 4 Director Facultad de Ciencias de la Salud, Universidad Anáhuac México, Cuidad de México 52786, Mexico; 5 Laboratorio de Medicina Genómica, Hospital Regional “Primero de Octubre”, ISSSTE, Av. Instituto Politécnico Nacional 1669, Lindavista, Gustavo A. Madero, Ciudad de Mexico 07300, Mexico

**Keywords:** childhood cancer, septicemia, multidrug-resistant (MDR) bacteria, extensively drug-resistant (XDR) bacteria, pandrug-resistant (PDR) bacteria, post-therapeutic neutropenic fever, Mexico

## Abstract

Death in cancer patients can be caused by the progression of tumors, their malignity, or other associated conditions such as sepsis, which is a multiphasic host response to a pathogen that can be significantly amplified by endogenous factors. Its incidence is continuously rising, which reflects the increasing number of sick patients at a higher risk of infection, especially those that are elderly, pediatric, or immunosuppressed. Sepsis appears to be directly associated with oncological treatment and fatal septic shock. Patients with a cancer diagnosis face a much higher risk of infections after being immunosuppressed by chemotherapy, radiotherapy, or anti-inflammatory therapy, especially caused by non-pathogenic, Gram-negative, and multidrug-resistant pathogens. There is a notorious difference between the incidence and mortality rates related to sepsis in pediatric oncologic patients between developed and developing countries: they are much higher in developing countries, where investment for diagnosis and treatment resources, infrastructure, medical specialists, cancer-related control programs, and post-therapeutic care is insufficient. This situation not only limits but also reduces the life expectancy of treated pediatric oncologic patients, and demands higher costs from the healthcare systems. Therefore, efforts must aim to limit the progression of sepsis conditions, applying the most recommended therapeutic regimens as soon as the initial risk factors are clinically evident—or even before they are, as when taking advantage of machine learning prediction systems to analyze data.

## 1. Introduction

The World Health Organization (WHO) defines cancer as the uncontrolled growth and spread of cells that can affect any tissue in the body. According to data from the WHO, during 2015, 1 out of 6 deaths worldwide was related to cancer, and the WHO predicts a 70% increase in cancer cases during the next two decades. Cancer is a condition mainly associated with populations above 50 years old (87% of cases) [1,2].

Death in cancer patients can be caused by the tumor progression, their malignity, or by other associated conditions (e.g., sepsis). Sepsis is particularly important, as it appears to be directly associated with oncology therapy and fatal septic shock [3]. According to Cara B. Thurman et al., patients with cancer diagnosis face a much higher risk of infections after being immunosuppressed by chemotherapy, radiotherapy, or anti-inflammatory therapy. Additionally, these high-risk treatments demand in-hospital administration, thus increasing the potential for nosocomial infections [4]. In this manner, severe complications decrease the poor survival rates for pediatric cancer patients.

Fever is the primary reason pediatric oncology patients present to the emergency services; some studies have revealed that 0.9%–39% of febrile nonneutropenic oncology patients are bacteremic, and it has been estimated that 7.3% of oncology patients who are admitted to emergency services have positive bacterial blood cultures. The factors that increase the risk of sepsis development include URI (upper respiratory infection), with an increase of sepsis risk by 2.3 times, neuroblastoma by 3.6 times, other cancer diagnoses by 4.5 times, and tunneled externalized CVC (central venous catheter) by 5 times.

The hallmark of immunosuppressed patients undergoing cancer treatment is neutropenia, defined as a granulocyte count below 500 cells per cubic millimeter [5]. When this white blood cell count is accompanied by episodes of fever above 38 °C, it is said that the patient suffers from a neutropenic fever. The empirical use of more powerful antibiotics and antifungals, as well as the improvement of post-therapeutic techniques, have reduced neutropenic fever mortality rates in children to 7% worldwide [6,7]; however, the emergence of multidrug-resistant pathogens still represents a challenge for immunosuppressed oncological patients, particularly increasing the infant mortality rate.

Here we provide an overview of antibiotic-resistant sepsis development during chemotherapy in the pediatric oncological patients with neutropenic fever, with emphasis on the nosocomial infections caused by multidrug-resistant (MDR), extensively drug-resistant (XDR), and to pandrug-resistant (PDR) bacteria.

## 2. Background

Although the association between cancer and infection is well known, few studies have been performed to determine how harmful it is. A study published in 2004 evaluated data from six states of the United States in order to establish a relationship between oncological hospitalizations and sepsis. Its results showed that sepsis is a very common and severe complication among oncological patients. They are three times more likely to develop it [8]; 4.9% of the 2.5 million cancer-related hospitalizations per year in the United States develop sepsis. Additionally, sepsis is responsible for 36.7% of mortality in hospitalized cancer patients, and 8.5% of the total cancer-related deaths.

Neutropenic fever’s etiological agents have changed over a long period of time. During the 1970s, Gram-negative bacteria were the most common pathogens involved with sepsis, however, starting in the 1990s there was a transition to Gram-positive bacteria—most commonly those belonging to *Staphylococcus*. Recently, the predominance of Gram-negative microorganisms has come back, mainly characterized by *Klebsiella* and *E. coli* strains [7]. These epidemiological changes are associated with different approaches used in patients with cancer, such as the prophylactic use of fluoroquinolones and the in-hospital use of intravascular catheters. Additionally, it should be noted that the increase of severe mucositis incidence is also associated with the use of chemotherapy [9].

## 3. Statistics and Socio-Economic Gaps

The Global Cancer Observatory’s 2014 statistics, belonging to the International Agency for Research on Cancer, were included in the 2015 World Cancer Report of the World Health Organization (WHO). They reported 14.1 million new cancer cases and 8.2 million deaths associated with cancer worldwide [10]. It has been predicted that a 70% increase in cancer incidence may occur over the next two decades [1,2].

In first-world regions such as North America and Western Europe, healthcare systems are equipped to detect and diagnose early cancer development, therefore skyrocketing cancer incidence. At the same time, cancer mortality rates decrease significantly within these regions, in comparison to developing countries with poor economies. Late diagnosis and insufficient oncological treatment care are associated with higher morality [1,2].

**Figure 1:**
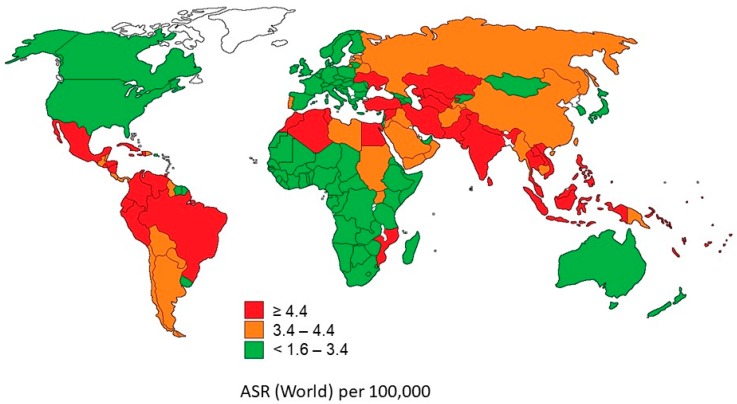
Estimated ASR (age-standardized mortality rates) World in 2018, all cancers, both sexes, age 0–19. Based on the International Agency for Research on Cancer, “Global Cancer Observatory.” https://gco.iarc.fr/ [13].

Underdeveloped countries reported an incidence of 57%, which represents 8 million new cases, and 65% mortality, which represents 5.3 million deaths annually (48% of these happened during the first 5 years after the diagnosis) [10]. According to the National Cancer Institute of the United States (part of the National Institutes of Health (NIH)), more than 60% of new cancer cases globally are found in Asia, Africa, and South and Central America, and 70% of all cancer deaths are concentrated in these regions (Figure 1) [11,12,13].

At present, 90% of developed countries’ healthcare systems include oncological treatments, while less than 30% of underdeveloped countries’ healthcare systems do so. In order for countries to improve their statistics within the oncological area, they must have trustable data to re-design their healthcare strategies; only one out of five low-income countries have this information. It should be noted that although post-treatment care is considered fundamental, in these countries only 14% of oncological patients have access to it [14]. The 90% of diagnosed children live in underdeveloped countries, where the chance of survival is less than 10%, in contrast to the 80% chance of survival registered in the developed countries [15].

Childhood cancer appears before the age of 15. Today, in developed countries, the 5-year survival rate is between 80%–90% of patients—a huge improvement when compared to the 30% reported 40 years ago. Investment on infrastructure and post-treatment care for pediatric oncologic patients has increased this survival rate by making available the means to reduce the cytotoxic impact of chemotherapy, immunosuppression, complications of hematopoietic stem cells transplantation (HSCT), radiotherapy, and aggressive surgical intervention.

It should be noted that in developing countries, young people represent more than 50% of the population, so, as estimated by the WHO report, these have a higher incidence of pediatric cancer [2]. According to the International Agency for Research on Cancer, between 2001 and 2010, approximately 300,000 cases of cancer were diagnosed in children and teenagers every year. About half of them die each year due to the severe complications of cancer [15].

## 4. Role of Post-therapeutic Oncological Support Services Unit

Multidisciplinary teams, consisting of doctors, nurses, social workers, children’s life specialists, schoolteachers, psychologists, religious experts, and others, are fundamental for the treatment of pediatric cancer. When we talk about post-therapeutic care, the role of nurses is of vital importance as they are considered the coordinators of the entire care of the patient. The staff of the intensive care unit must be focused on preventing risks threatening patients’ lives. They should count on adequate facilities and highly specialized staff in the post-therapeutic care of pediatric oncology patients.

The WHO remarks on the importance of national cancer control programs, defined as “public health programs designed to reduce the number of cancer cases, deaths and improve life quality of cancer patients, by implementing systematic, equitable and evidence-based strategies for prevention, early detection, diagnosis, treatment and palliation using available resources”. According to data, “equitable” is the characteristic of these programs which is least attended, as low-income people do not have access to the latest therapeutic technologies, and sometimes, to adequate post-therapeutic care [16].

According to the report “Cancer Control Access and Inequality in Latin America: A Tale of Light and Shadow” by the Economist’s Intelligence Unit [17], currently in Latin America a vast amount of people have access to cancer treatment as never before; however, it is not yet enough due to very limited resources in specific areas (e.g., rural regions), the increasing phenomenon of “medical apartheid” that is restricting poorer citizens to less well-resourced care, and a widespread lack of palliative care. This report concludes that huge investment in the public sector to boost national cancer control programs is fundamental for Latin American countries. According to the WHO, in order for a national healthcare system be sustainable, it requires at least 6% of the nation’s GDP; however, in Latin American countries, the percentage of GDP allocated to medical care is less than half, which is clearly insufficient to fight the emerging crisis that cancer represents within them [17].

In Mexico, the National Health Protection Commission (Comisión Nacional de Protección en Salud) reports 5000– 6000 new pediatric cancer cases yearly; 50%–60% of these are diagnosed and treated in public institutions. The diagnosis of this disease is devastating, as it implies an important economic load, which is in many cases untenable for low-income families. The treatment costs not only imply the administration of oncologic drugs, but also the care for the patient for three years, plus two more years under surveillance. Additionally, transportation, lodging, feeding, laboratory tests, imaging, and supplementary medicines entail important costs to be considered [18,19,20,21].

The Mexican National Health Protection Commission reported that between 2013 and 2017, 11,725 treatments for childhood cancer cases were funded by this insurance, by means of a US$37.5 billion investment. On average, $8000 USD was invested in each case; however, this amount was not sufficient, as in 2015 the Economic Analysis Unit of the Health Secretary estimated the average cost for childhood cancer treatment doubled to USD$16,000. Taking these data into consideration, an investment above USD$8000 is required in addition to the financial aid provided by the Fund for Protection against Catastrophic Expenditures (FPGC) in order offer pediatric cancer patients the treatment and care needed [18,19,20,21].

Some challenges faced in providing adequate attention to oncological patients are the lack of resources for diagnosis and treatment, insufficient space for covering patients’ demands, the shortage of appropriate radiation equipment, and insufficient resources for bone marrow transplants and oncology-specialized units. Another challenge is the lack of medical specialists, such as pediatric oncologists, radiotherapists, pediatric surgeons, or hematologists, which do not belong to the core staff of many institutions, limiting the possibilities of treatment and better prognosis. About this last, in Mexico there are 56 Accredited Medical Units (AMUs) for oncological medical care in children under 20 years of age, and 10.7% (*n* = 6) of them possess the infrastructure capacity to offer therapy for malignant hematopathies, solid tumors of the central nervous system (CNS), solid tumors outside the central nervous system (CNS), and bone marrow transplant; 64.3% (*n* = 36) offer three of these services, 8.9% (*n* = 5) offer two services, and 16.1% (*n* = 9) offer only one service. The oncology service for malignant hematopathies is offered in 96.4% (*n* = 54) of AMUs, therapy for CNS solid tumors is offered in 73.2% (*n* = 41) of AMUs, treatment for solid tumors outside of CNS is offered in 83.9% (*n* = 47), and only 16.1% (*n* = 9) of all AMUs offer bone marrow transplant. All of these services are offered by 165 pediatric oncologists, 35 pediatric oncology surgeons, 36 pediatric hematologists, and 10 pediatric radiotherapists, which are clearly insufficient to meet the high demand. The lack of integral service observed in the remaining 50 units (89.3%) represents an area of growth opportunity for the country [18,19,20,21].

Of all their patients, 12% abandon treatment; 4.69% do so during the first year. In remote states or communities, away from medical attention centers, the treatment interruption increases up to 18%—5.67% during the first year, leading to a huge fall in patient survival [18,19,20,21].

Recently, Mexican authorities established a comprehensive program for the prevention and control of cancer; however, the limited federal budget makes it impossible to be fully implemented before 2019. In this manner, it should be noted that the short budget and its incorrect assignment, the ineffective collaboration between governmental and non-governmental organizations, the health professionals’ instability, and the lack of effective health information systems drastically reduce patient survival [18,19,20,21].

## 5. Multidrug-Resistant Post-Therapeutic Infections

Febrile neutropenia is characterized by oral temperature ≥38.5 °C (or two consecutive readings of ≥38.0 °C with a space of 1 hour) and absolute neutrophil count of ≤500 cells/μL. This condition is very common in pediatric oncological patients, and is mainly associated with post-chemotherapy infection, more specifically with the development of sepsis [22,23].

Unfortunately, the condition of the patient, as well as the prognosis and treatment, will worsen in relation to the antimicrobial resistance profile of the bacteria associated with the infection [24,25].

Simmon et al. (2005) performed the first multicenter prospective study confirming that pediatric oncology patients have a much higher risk of developing an infectious condition. They reported that septicemia was mainly caused by venous catheter contamination. Among the etiological agents associated with these infections are *Staphylococcus aureus*, coagulase-negative staphylococci (CoNS), *Klebsiella* spp., *Pseudomonas aeruginosa*, *Escherichia coli*, *Enterobacter cloacae*, *Enterococcus faecium* and *K. pneumoniae*. In some cases, multiple infections (infections produced by more than one bacterium) were reported, and many of them were due to multidrug-resistant bacteria [26].

In 2005, a study done in Israel showed the trends in the microbiological spectrum of pediatric febrile oncologic patients when treated with two different empirical antibiotic regimes: (1) ceftazidime and gentamicine; (2) piperacillin/tazobactam and amikacin. Results showed that 81 bacteremia episodes occurred in just 41 patients; 132 microorganisms were isolated, of which 84 (65%) were Gram-negative bacteria, 39 (30%) were Gram-positive bacteria, and 7 (5%) were fungi. When looking for antibiotic resistance, 5 (18%) out of 28 *E. coli* and *Klebsiella* spp. isolates were β–lactamase producers, while no methicillin-resistant microorganisms were detected. Both antibiotic regimes were effective, but better results were achieved with the second one (piperacillin/tazobactam and amikacin) [27].

Another study carried out in Italy (2005) showed interesting results. The proportion of Gram-negative ciprofloxacin-resistant bacteria isolated in the blood cultures of oncological pediatric patients that had not received antibiotic prophylaxis was 10% in a pediatric hospital from Genoa, where the fluoroquinolone use was very restricted, in comparison to one in Rome where this proportion reached 41% when fluoroquinolone use was common. In addition, the simultaneous resistance to ciprofloxacin, ceftazidime, amikacin, and imipenem/cilastatin was 11% in Genoa, while it was 37% in Rome. The resistance to ciprofloxacin was higher in children that had a shared environment with adults receiving prophylactic therapy. The study concluded that the use of fluoroquinolones as prophylactic agents can lead to the development of infections by multidrug-resistant bacteria, and also that their use is a contraindication in pediatric patients due to their adverse effects. The isolated microorganisms were: 54 *Pseudomonas aeruginosa* strains, 32 other *Pseudomonadaceae*, 62 *Escherichia coli* strains, 63 other members of the *Klebsiella–Enterobacter–Serratia* group, 49 other Gram-negative bacilli and 174 Staphylococci, 39 *Staphylococcus aureus* strains, and 135 coagulase-negative Staphylococci strains [28].

Yet, another study done during 2005 in the United States reported that children with leukemia treated with hematopoietic stem cell transplants and treated preventively with antibiotics later developed infections. From 184 patients, 74 (41%) developed bacteremia. The microorganisms responsible were mainly Gram-positive cocci, *Staphylococcus* (50%) and *Streptococcus* (28%) being the most common. Gram-negative microorganisms were isolated from 22% of patients, including *Pseudomonas* strains (5.7%) and *Klebsiella* species (3.4%). From streptococcal infections, 72% showed resistance to ampicillin, and only 25% of Gram-negative bacteria were resistant to gentamicin [29].

In another multicenter study done in Italy between 2000 and 2008, results were obtained on the prevalence of resistant *Pseudomonas aeruginosa* strains, which is the main etiological agent of nosocomial infections. The presence of these bacteria was evaluated in 12 pediatric oncology centers. Results showed that 31.4% of isolates were MDRPA (multidrug-resistant *Pseudomonas aeruginosa*). Death within 30 days of patients with positive blood culture occurred in 19.6% (25/127) of total patients; infections by MDRPA occurred in 35.8% of patients (14/39) [30].

A study conducted during 2007 in Malaysia described isolates mainly from Gram-positive microorganisms obtained after treating oncologic pediatric patients with chemotherapy. Specifically, the study mentioned coagulase-negative microorganisms, which had high rates of resistance to methicillin (76% of strains) [31].

A systematic analysis conducted between 2006 and 2011 in Italy concluded that there were significant differences within centers that developed studies on bacteremia etiologies and drug-resistance prevalence, with patterns that changed over time. Altogether, an overall decrease in the Gram-negative to Gram-positive relation was observed, together with an increase in resistance rates—particularly within enterobacteria. The increase of resistance is critical in the case of Gram-negative pathogens, as very few new antibiotics against these microorganisms are expected. As a result, considering the important differences between centers and the possibility of a quick change, a deep knowledge of the local epidemiology is extremely important to guide decision-making on optimal empirical therapy considering pediatric patients’ condition. This transition towards a predominance of multidrug-resistant Gram-negative microorganisms was confirmed by another study conducted in Spain during 2012, highlighting the clinical challenge that facing these microorganisms represents [32].

In Sweden, Ola Blennow et al. described the epidemiological and clinical implications of the increasing frequency of multidrug-resistant microorganisms in patients undergoing treatment against hematologic pathologies. Two important changes in the epidemiology of bloodstream infections during the past decade have been the increasing prevalence of Gram-negative microorganisms and the rapid increase in antimicrobial resistance within Gram-negative and Gram-positive isolates. These changes in resistance rates also include a greater detection of extended spectrum β-lactamases (ESBLs) in Enterobacteriaceae that even get to produce carbapenemases. In addition, multidrug-resistant *Pseudomonas aeruginosa* and *Acinetobacter* spp., methicillin-resistant *Staphylococcus aureus* (MRSA), and vancomycin-resistant enterococci (ERV) strains have been isolated. These results are global threats, but intensive care patients as well as immunosuppressed oncologic patients suffer the greatest damages [33].

Experts in the field of antimicrobial resistance in joint work with the European Center for the Prevention of Diseases and Control (ECDC) and the Centers for Disease Control and Prevention (CDC) were able to establish the limits and definitions among resistant bacteria: multidrug-resistant (MDR), extensively drug-resistant (XDR), and to pandrug-resistant (PDR) bacteria [34].

This was based on the acquired resistance profiles of the main resistant bacteria frequently associated with infections associated with medical care. These are *Staphylococcus aureus*, *Enterococcus* spp., *Enterobacteriaceae* (other than *Salmonella* and *Shigella*), *Pseudomonas aeruginosa*, and *Acinetobacter* spp. [34].

For each bacterium, epidemiologically significant antimicrobial categories were established. These categories were established based on the documents and cut-off points of the Clinical Laboratory Standards Institute (CLSI), the European Antimicrobial Sensitivity Testing Committee (EUCAST), and the United States Food and Drug Administration (FDA) [34].

The following definitions were established: Multidrug-resistant bacteria (MDR) possess acquired resistance to at least one antibiotic of three or more categories. Extensively drug-resistant (XDR) bacteria possess resistance to at least one antibiotic of almost all categories, except one or two of them. Pandrug-resistant bacteria (PDR) are resistant to all agents of all categories of antimicrobials [34].

## 6. A Brief Look at the Pathophysiology of Sepsis

**Figure 2:**
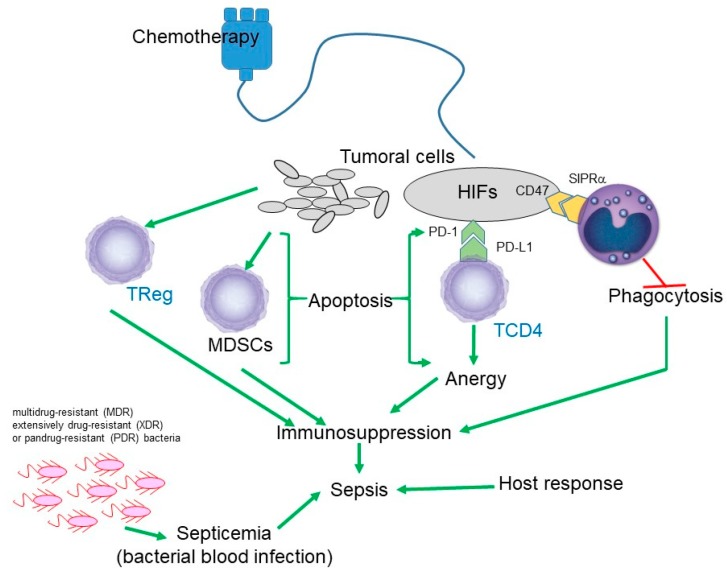
Schematic representation of the immunosuppressive effect produced both by the cancer cells themselves and by chemotherapy. HIF: hypoxia-inducible factor; MDSC: myeloid-derived suppressor cell; PD1: programmed cell death-1; PDL1: programmed cell death-1 ligand; SIRPα: signal-regulatory protein alpha; TReg: regulatory T cell; TCD4: T lymphocyte CD4 or helper.

The phenomenon of immunosuppression has been widely studied in cancer disease. It has been described that tumor cells are able to induce the activation of various immunosuppressive pathways, one of which is the network described by Alizadeh and Larmonier in 2014, where regulatory T lymphocytes (Tregs) and myeloid-derived suppressor cells (MDSCs) participate, which will inhibit the activity of dendritic cells, NK cells, CD8 + CTLS cells, CD4 + Th cells, and δγ T cells (Figure 2) [35].

On the other hand, it has been described that chemotherapy by itself is able to induce immunosuppression. Samanta et al. in 2018 described that chemotherapy induces the production of hypoxia-inducible factors (HIFs) in tumor cells. HIFs activate CD47, which is a ligand of signal-regulatory protein alpha (SIRPα) in the macrophage with the consequent inhibition of phagocytosis. HIF also activates the expression of programmed cell death-1 ligand (PDL1) which binds to PD1, inducing the anergy of T Cells (Figure 2) [36].

Immunosuppression associated with infection becomes more critical if it is due to a multidrug-resistant (MDR) or extensively drug-resistant (XDR) bacterium, and it results in a worse prognosis if it is due to pandrug-resistant (PDR) bacteria. The immunosuppression observed in cancer patients represents an important risk factor for developing sepsis, leading to higher mortality rates and higher costs for the healthcare sector.

**Figure 3:**
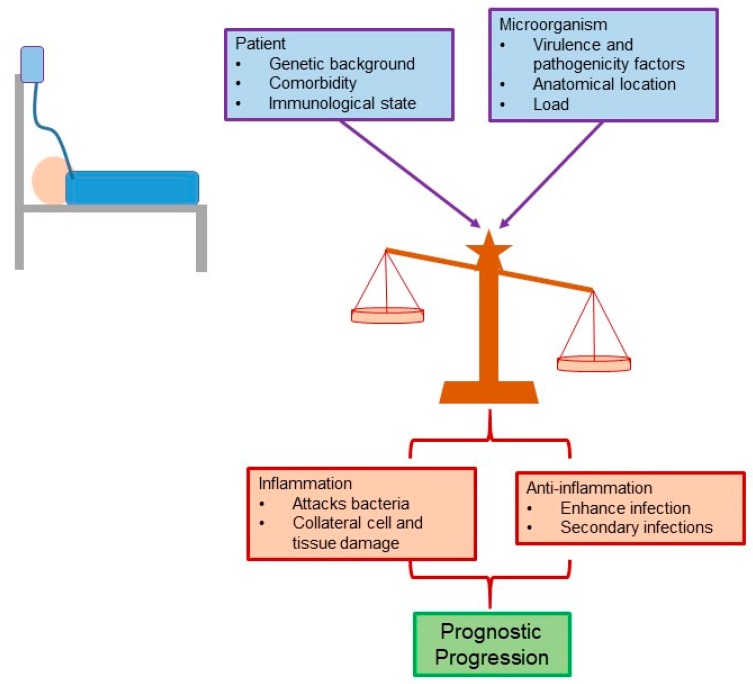
The clinical characteristics and pathophysiology of sepsis are multifactorial in nature.

For a long time, the main cause of the clinical picture presented during sepsis (systemic inflammatory response syndrome, SIRS) was attributed to the excessive inflammatory response. However, recent evidence points to the fact that the clinical characteristics and the pathophysiology of sepsis are multifactorial in nature, and the intrinsic characteristics of the patient (e.g., comorbidity) and their genetic load should also be considered. In this way, the characteristics of the microorganism(s) involved, such as the bacterial load and the factors of pathogenicity and virulence, also influence this disease (Figure 3) [37].

Inflammatory signals designed to eliminate the microorganism are associated with tissue and organ collateral damage. On the other hand, anti-inflammatory signals lead to an exacerbation of the infection and the possible participation of secondary infections. Therefore, the balance between these two mechanisms—inflammatory and anti-inflammatory—directly affects the length of stay, disease severity, and prognosis of the patient (Figure 3) [38].

In sepsis, like many bacterial infections, there is an interaction between microorganisms and the cells of the immune system. In this interaction, bacteria participate in structures conserved across microbial species—so-called pathogen-associated molecular patterns (PAMPs). One of the most common PAMPs is lipopolysaccharide (LPS), which is present in Gram-negative bacteria [39].

On the other hand, the cells of the immune system recognize these PAMPs by means of receptors such as Toll-like receptors (TLRs), retinoic acid-inducible gene-I-like receptors (RIG-I-like receptors), C-type lectin receptors (CLRs), and NOD-like receptors (NLRs) [40].

The injury and death of various cell types occurs during the defensive process against microorganisms, and with it, the liberation of the endogenous molecules of injured or dead cells. These molecules have been called damage-associated molecular patterns (DAMPs). Among the most studied DAMPs are the B1 protein of the high mobility group, the S100 proteins, and the RNA, DNA, as well as extracellular histones [39].

When DAMPs are recognized by the cells of the immune system, they initiate the liberation of a complex of chemical mediators, such as proinflammatory cytokines, complement system, platelet activating factor, arachidonic acid metabolites, nitric oxide (NO), and reactive oxygen species (ROS) [39].

On the other hand, bacterial exotoxins called superantigens, such as the erythrogenic toxin of *Streptococcus pyogenes* or the toxin associated with toxic shock of *Staphylococcus aureus*, can create a kind of bridge between the major histocompatibility complex class II (MHC-II) of the antigen-presenting cells (APCs) for example dendritic cells and T-cell receptors (TCRs) in ThCD4 lymphocytes. This bridge promotes the non-specific binding of MHC-II and its non-corresponding TCR, which will generate an equivocal production of cytokines and lead to a cytokine cascade or storm [41].

The activation of the complement system, the cytokine storm, the activation of arachidonic acid and the synthesis of prostaglandins, and especially the release of nitric oxide and reactive oxygen species, will generate collateral tissue and cellular damage. This damage is associated with the generalized activation of coagulation factors, which will induce generalized coagulation with the consequent depletion of coagulation factors and the subsequent presentation of generalized edema and hemorrhages and alteration of the blood perfusion, a state known as disseminated intravascular coagulation (DIC) [42,43].

**Figure 4:**
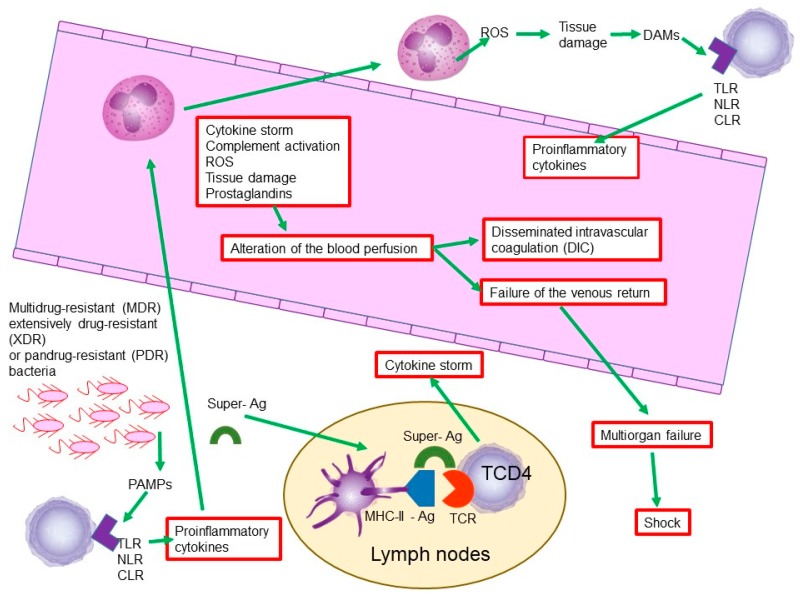
A brief look at the molecular and cellular mechanisms involved in the pathophysiology of sepsis. CLR: C-type lectin receptor; DAMP: damage-associated molecular pattern; MHC-II: histocompatibility complex class II; NLR: NOD-like receptor; PAMP: pathogen-associated molecular pattern; ROS: reactive oxygen species; TCR: T-cell receptor; TLR: Toll-like receptor.

The tissue damage and the alteration of the blood perfusion leads to the failure of the venous return, which generates multiorgan failure, contributing to a worse prognosis of the state of sepsis that dangerously derives the state of shock (Figure 4) [42,43].

The state of sepsis becomes even more critical and of worse prognosis in immunosuppressed patients, due to its inability to generate an adaptive immune response against these microorganisms. However, this situation becomes even more dangerous in these patients when the bacteria involved turn out to be multidrug-resistant (MDR) or extensively drug-resistant (XDR), and it is fatal if they are pandrug-resistant (PDR) [44]. Sepsis is currently defined a multiphasic host response to a pathogen that can be significantly amplified by endogenous factors. Previously, sepsis was considered as an infectious condition with at least two out of four systemic inflammatory response syndrome (SIRS) criteria [37]. However, it has been observed that most patients entering the intensive care unit have at least two criteria without having sepsis. Thus, it is now considered that sepsis involves an early activation of pro- and anti-inflammatory responses along with important modifications to non-immunological pathways such as cardiovascular, neural, autonomous, hormonal, metabolic, and coagulation pathways, which are part of the prognosis [37].

Currently, there are several scales used as sepsis severity calculators, which allow the identification of optimal treatments according to the stage of the condition. They establish prognosis through validated mathematical calculations, evaluating the quality and efficiency of intensive care units. In addition, they correlate with mortality and in-hospital stay [37,45]. One of the most frequently used in the hospital environment is the Sequential Organ Failure Assessment (SOFA) [46]. The predictive validity of the SOFA score for in-hospital mortality among critically ill patients with suspected sepsis is higher than that of the SIRS criteria [47]. In addition, it should be noted that the SOFA score evaluates clinical and biochemical parameters in order to determinate the condition’s severity and the need for quick and appropriate intervention, if it has not been yet established [37].

In a meta-analysis of the 30 most important studies on multi-organ failure from the past decades, the ideal criteria as a detector of dysfunction in each organ are described. Four scales have been proposed for multi-organ failure, all of them including all six systems: cardiovascular, respiratory, liver, hematopoietic, renal, and neurological. This has several advantages: (1) it results from the physiological alterations of six organs; (2) it correlates with mortality since day one; (3) it shows that mortality correlates with the number of failed organs and their degree of dysfunction; (4) it shows early anomalies as well as changes after admission.

Septic shock is a condition where circulatory, cellular, and metabolic anomalies are deep enough to substantially increase in-hospital mortality. This condition is defined as sepsis complicated by either hypotension that is refractory to fluid resuscitation or by hyperlactatemia [48]. Patients in septic shock can be identified by a clinical construction of sepsis with persistent hypotension that requires vasopressors to keep mean arterial pressure at 65 mmHg and lactate levels >2 mmol/L (18 mg/dL) in spite of an adequate reanimation. The incidence of sepsis is continuously increasing, which reflects the increasing numbers of sick patients at higher risk of infection, especially the elderly, pediatric, and immunosuppressed ones [37]. Several studies show that sepsis is frequently associated with initial foci of infection in the lungs mainly and to a lesser extent with urinary infections. [48].

This infection takes high relevance when it is caused by drug-, multidrug-, or pandrug-resistant nosocomial bacteria, and when it is caused by medical procedures or long in-hospital stay.

## 7. Big Data Analysis to Predict or Identify Sepsis Development

The term “big data” refers to information collected on a large scale and evaluated to obtain new insights or forms of usefulness [49]. The growing evolution of big data analysis techniques makes it necessary to develop platforms capable of analyzing perioperative risks dynamically and in real time, starting from large amounts of newly generated information or that stored in electronic clinical files. This data is characterized by its high dimensionality, sparsity, and heterogeneity. Data-driven deep-learning approaches, also known as DNNs (deep neural networks), are methods to approximate parametric functions by using algorithms and training data [50].

New diagnostic methods developed based on automatic learning algorithms for the analysis of big data obtained from pediatric patients with serious illness have allowed us to differentiate between sepsis and the systemic inflammatory response syndrome (SIRS)—two common conditions that affect children with cancer. Both pathologies have similar pathophysiological and clinical patterns, but different causes. With this approach, it has been possible diminish the risk of mistreatment in actual septic cases and to reduce 20.6% of the in-hospital stay time and 12.4% of in-hospital mortality [51,52,53].

## 8. Conclusions

Childhood cancer is a serious global health problem that is on the rise, especially affecting underdeveloped countries, which have the highest incidence of the disease and the worst prognosis of survival. In this matter, one of the great obstacles faced by underdeveloped countries is the post-therapeutic management of the patient. After chemotherapy, and as a secondary effect of it, the patient suffers from immunosuppression. This generates a critical period of susceptibility to infectious processes. Although in many cases childhood cancer can be curable, one of the main causes of death is due to the development of sepsis, mainly associated to post-therapeutic neutropenia. This condition is worsened when the etiological agents responsible are multidrug-resistant bacteria—especially Gram-negative ones.

The uncontrolled widespread use of antibiotics, mainly ciprofloxacin and ceftazidime, amikacin, or imipenem/cilastatin in immunosuppressed patients, have induced a high selection pressure on the microorganisms and have been the driving force for the changes in the epidemiology of pathogens. Currently, mainly Gram-negative pathogens such as *Klebsiella* and *E. coli* are found in oncologic pediatric patients with sepsis. However, there are no studies on Latin American populations identifying the accurate etiology of sepsis among these patients. The treatment of cancer should be multi-disciplinary, with emphasis on intensive therapy to prevent and decrease the risk of new or recurrent infections.

Considering the current pediatric cancer control studies, even if healing and detection rates have increased, this trend only is applicable for developed countries; underdeveloped countries lack or do not have access to the advanced technologies used in prevention, early child cancer detection, and the post-therapeutic management of cancer patients. One of the clear strategies that must be implemented by the healthcare sector is adequately distribution the necessary resources to this condition, in order to facilitate the early detection and efficient prevention of sepsis-associated complications.

A second strategy to be considered is increasing the financing of new specialists in pediatric oncology and medical centers, including the incorporation of new medical personnel and adequate academic training to optimize treatments and post-therapeutic care, increasing survival and quality of life for oncologic pediatric patients.

Starting from the premise that prevention is always better than treatment, it is much more practical to take post-therapeutic care measures than to treat patients with a severe sepsis event with a reserved prognosis. As part of the strategies to reduce mortality from this disease, the countries involved must invest in the research and monitoring of multidrug-resistant pathogens.

There are no studies within the Mexican population that estimate sepsis incidence in childhood patients with a cancer diagnosis. However, a significant association between immunosuppression and the frequent development of sepsis in these patients has been established. New studies should be undertaken to clarify the pathology of childhood cancer and its treatment as a risk factor for developing new infectious entities or sepsis.

Machine-learning models must be considered as an alternative to ensure timely diagnosis and antibiotic treatment of sepsis in pediatric oncologic patients during post-therapeutic care, increasing survival rates and reducing sepsis-related costs.

Even if immunosuppression in pediatric patients has been associated with sepsis development, morbidity and mortality statistics can be yet improved, along with patient quality of life, by the creation and optimization of multidisciplinary teams focused on prevention, treatment, and post-therapeutic care. Their efforts must aim to limit the progression of sepsis conditions, applying the most recommended therapeutic regimens even before the initial risk factors are clinically evident.
